# Current State of Coal Fly Ash Utilization: Characterization and Application

**DOI:** 10.3390/ma16010027

**Published:** 2022-12-21

**Authors:** Dmitry Valeev, Alex Kondratiev

**Affiliations:** 1 Laboratory of Sorption Methods, Vernadsky Institute of Geochemistry and Analytical Chemistry, The Russian Academy of Sciences, 119991 Moscow, Russia; 2 Scientific Research Centre “Thermochemistry of Materials”, National University of Science & Technology “MISIS”, 119049 Moscow, Russia

## 1. Introduction and Scope

This Special Issue of *Materials* is devoted to various aspects of coal fly ash (CFA) utilization. The Guest Editors of the issue were Dr. Alex Kondratiev and Dr. Dmitry Valeev. The Special Issue was designed to include submissions for characterization, mineralogy, hydrometallurgy, recovery of rare earth elements, Al and Si extraction, alkali and acid leaching, thermodynamics, recycling and solid waste minimization, leaching kinetics, autoclaved hydration, mechanical performance, polymerization, characterization of tensile strength and barrier deformation, physicochemical properties, and energy absorption capacity. Suggested application areas include construction and building industry and non-ferrous metallurgy. Twelve high-quality peer-reviewed articles from Russia, China, Czech Republic, Poland, USA, and France were selected and accepted for publication.

## 2. Contributions

Zhang et al. [1] submitted an article entitled “Circulating Fluidized Bed Fly Ash Mixed Functional Cementitious Materials: Shrinkage Compensation of f-CaO, Autoclaved Hydration Characteristics and Environmental Performance”. Circulating fluidized bed (CFB) fly ash is a by-product of CFB power generation that is hard to utilize in cement because it contains f-CaO and SO_3_. This work aims to explore the mechanism of the shrinkage compensation of free-CaO (f-CaO) and the autoclaved hydration characteristics and environmental performance of CFB fly ash mixed with cementitious materials (CMM). In this work, the long-term volume stability of CMM is improved with the addition of CFBFA. These findings suggest that the compressive strength of sample CMM0.5 is the highest under both standard conditions (67.21 Mpa) and autoclaved conditions (89.56 Mpa). Meanwhile, the expansion rate (0.0207%) of sample CMM0.5 is the lowest, which proves the shrinkage compensation effect of f-CaO in CFBFA. The main hydration products of CMM0.5 are Ca_2_SiO_4_·H_2_O (C-S-H) gel, CaAl_2_Si_2_O_7_(OH)_2_·H_2_O (C-A-S-H) gel, and Ca(OH)_2_. In addition, the high polymerization degree of [Si(Al)O_4_] and the densified microstructure are presented in the sample CMM0.5. The leaching results indicate that the heavy metals in CMM0.5 satisfy the WHO standards for drinking water due to physical encapsulation and charge balance. Therefore, this investigation provides a novel method of using CFB fly ash in cement.

El Mendili et al. [2] contributed an article entitled “Mud-Based Construction Material: Promising Properties of French Gravel Wash Mud Mixed with Byproducts, Seashells and Fly Ash as a Binder”. The French gravel industry produces approximatively 6.5 million tons of gravel wash mud each year. This material offers very promising properties, which require an in-depth characterization study before its use as a construction material; otherwise, it is removed from value cycles by disposal in landfills. We examined the suitability of gravel wash mud and seashells, with fly ash as a binder, as an unfired earth construction material. Thermal and mechanical characterizations of the smart mixture composed of gravel wash mud, Crepidula fornicata shells, and fly ash are performed. The new specimens exhibit high compressive strengths compared to usual earth construction materials, which appears as a good opportunity for a reduction in the thickness of walls. The use of fly ash and Crepidula shells in addition to gravel wash mud provides high silica and calcium contents, which both react with clay, leading to the formation of tobermorite and Al-tobermorite as a result of a pozzolanic reaction. Considering the reduction in porosity and improvements in strength, these new materials are good candidates to contribute significantly to the Sustainable Development Goals (SDGs) and reduce carbon emissions.

Wang et al. [3] provided an article entitled “Effect of Electrolytic Manganese Residue in Fly Ash-Based Cementitious Material: Hydration Behavior and Microstructure”. Electrolytic manganese residue (EMR) is a solid waste with a main mineralogical composition of gypsum. It is generated in the production of metal manganese by the electrolysis process. In this research, EMR, fly ash, and clinker were blended to make fly ash-based cementitious material (FAC) to investigate the effect of EMR on strength properties, hydration behavior, microstructure, and environmental performance of FAC. XRD, TG, and SEM studied the hydration behavior of FAC. The pore structure and [SiO_4_] polymerization degree were characterized by MIP and ^29^Si NMR, respectively. The experimental results indicate that FAC shows excellent mechanical properties when the EMR dosage is 10%. Moderate content of sulfate provided by EMR can promote the hydration reaction of FAC, and it shows a denser pore structure and higher [SiO_4_] polymerization degree in this case. Heavy metal ions derived from EMR can be adsorbed in the hydration products of FAC to obtain better environmental properties. This paper presents an AFt covering model for the case of excessive EMR in FAC, and it importantly provides theoretical support for the recycling of EMR in cementitious materials.

Shoppert et al. [4] submitted an article entitled “Kinetics Study of Al Extraction from Desilicated Coal Fly Ash by NaOH at Atmospheric Pressure”. The most promising source of alumina in the 21st century is the coal fly ash (CFA) waste of coal-fired thermal plants. The methods of alumina extraction from CFA are often based on pressure alkaline, acid leaching, or preliminary roasting with different additives followed by water leaching. The efficiency of the alumina extraction from CFA under atmospheric pressure leaching is low due to the high content of acid-insoluble alumina phase mullite (3Al_2_O_3_·2SiO_2_). This research for the first time shows the possibility of mullite leaching under atmospheric pressure after preliminary desilication using high liquid-to-solid ratios (L:S ratio) and Na_2_O concentrations. The analysis of the desilicated CFA (DCFA) chemical and phase composition before and after leaching was carried out by inductively coupled plasma optical emission spectrometry (ICP-OES) and X-ray diffraction (XRD). The morphology and elemental composition of solid product particles were investigated by scanning electron microscopy with energy-dispersive X-ray spectroscopy (SEM-EDX). An automated neural network and a shrinking core model (SCM) were used to evaluate experimental data. The Al extraction efficiency from DCFA was more than 84% at T = 120 °C, leaching time was 60 min, the L/S ratio > 20, and concentration of Na_2_O was 400 g L^−1^. The kinetics analysis by SCM shows that the surface chemical reaction controls the leaching process rate at T < 110 °C, and, at T > 110 °C after 15 min of leaching, the process is limited by diffusion through the product layer, which can be represented by titanium compounds. According to the SEM-EDX analysis of the solid residue, the magnetite spheres and mullite acicular particles were the main phases that remained after NaOH leaching. The spheric agglomerates of mullite particles with non-porous surface were also found.

Hwang and Yeon [5] contributed an article entitled “Fly Ash-Added, Seawater-Mixed Pervious Concrete: Compressive Strength, Permeability, and Phosphorus Removal”. A mixed proportion of off-spec fly ash (FA)-added and seawater-mixed pervious concrete (SMPC) was optimized for compressive strength and permeability, and then the optimized SMPC was tested for the rate and extent of aqueous phosphorus removal. An optimum mix proportion was obtained to attain the percentages (%wt.) of FA-to-binder at 15.0%, nano SiO_2_ (NS)-to-FA at 3.0%, liquid-to-binder at 0.338, and water reducer-to-binder at 0.18%, from which a 7-day compressive strength of 14.0 MPa and a permeability of 5.5 mm/s were predicted. A long-term maximum compressive strength was measured to be ~16 MPa for both the optimized SMPC and the control ordinary pervious concrete (Control PC). The phosphorus removal was favorable for both the optimized SMPC and the Control PC based on the dimensionless Freundlich parameter (1/n). Both the optimized SMPC and Control PC had a first-order phosphorus removal constant of ~0.03 h^−1^. The optimized SMPC had a slightly lower capacity of phosphorus removal than the Control PC based on the Freundlich constant, Kf (mg^1−1/n^ kg^−1^ L^1/n^): 15.72 for the optimized SMPC vs. 16.63 for the control. This study demonstrates a cleaner production and application of off-spec FA-added, seawater-mixed pervious concrete to simultaneously attain water, waste, and concrete sustainability.

Yatsenko et al. [6] provided an article entitled “Improving the Properties of Porous Geopolymers Based on TPP Ash and Slag Waste by Adjusting Their Chemical Composition”. The possibility of improving the properties of porous geopolymer materials based on ash and slag waste from thermal power plants by adjusting their chemical composition is considered. An X-ray phase analysis of ash and slag wastes was carried out, the geopolymers’ precursor compositions were calculated, and additives to correct their chemical composition were selected. The samples were synthesized, and their physical and mechanical properties (density, porosity, compressive strength, and thermal conductivity) were analyzed. The micro- and macrostructure of the samples and the pore distribution of the obtained geopolymers were studied, and the pore-distribution histograms were obtained. The influence of Si:Al ratio on structural changes was described. The geopolymers’ phase composition was studied, consisting of an amorphous phase and high quartz and mullite. A conclusion about the applicability of this method for obtaining high-quality porous geopolymers was made.

Wasil and Zabielska-Adamska [7] submitted an article entitled “Tensile Strength of Class F Fly Ash and Fly Ash with Bentonite Addition as a Material for Earth Structures”. The behavior of soils under tensile stress is of interest to geotechnical engineers. The tensile strength of soils is often associated with tensile fractures that can generate a privileged flow path. The addition of bentonite improves the plastic properties of the soil; therefore, the study was conducted for the compacted class F fly ash and fly ash with various ben-tonite additions. The amounts of bentonite were 5, 10 and 15%, calculated in weight relation to the dry mass of samples. The tensile strength of compacted clay was also established for comparison. Laboratory tests were carried out using the direct method (breaking) on cylindrical samples and the indirect method (the Brazilian test) on disc-shaped specimens. For this purpose, a universal testing machine with a frame load range of ±1 kN was used. It is stated that bentonite considerably influences the tensile strength of the fly ash evaluated with both methods. The tensile strength values obtained with the Brazilian method are comparable to or higher than those obtained with the direct method. The achieved tensile strength values of compacted fly ash, improved by 10–15% of bentonite addition, are comparable with the results obtained for clay used in mineral sealing, while the strain at maximum tensile strength is similar in the direct test and lower in the indirect test.

Šulc et al. [8] contributed an article entitled “A Study of Physicochemical Properties of Stockpile and Ponded Coal Ash”. The article describes chemical and also selected physical properties of ponded high-temperature fly ash (FA) and bottom ash (BA) from a Mělník lignite power plant located in the Czech Republic. The research was carried out on samples obtained from drills with a depth of up to 54 m and the age of the samples retrieved from the lowest layers of the stockpile dating back to 1960. At the same time, a comparison was made with fresh fly ash and fresh bottom ash obtained from the same power plant. The study analyzed a total of 98 stockpile samples. The properties selected were studied across the entire stockpile, namely moisture content, specific density, specific surface, carbon content, elemental and phase composition, pH, electrical conductivity and leachability. SEM analyses were also performed. The performed measurements of chemical properties proved the chemical stability of the material even after several decades of storage in the stockpile. The largest changes are evident in the results of the analyses related to the leachability of SO_3_, Cl^−^, and F^−^. In contrast, the pH does not change significantly, and the composition is pH neutral or alkaline. Regarding ponded BA, particle disintegration was noted depending on the increasing core borehole depth.

Li et al. [9] provided an article entitled “The Triaxial Test of Polypropylene Fiber Reinforced Fly Ash Soil”. Recently, soil reinforcement using arranged or randomly distributed fibers has attracted increasing attention in geotechnical engineering. In this study, polypropylene (PP) fibers with three lengths (6, 12, and 24 mm) and three mass percentages (0.5%, 1%, and 1.5%) were used to reinforce a coal fly ash soil (FAS) mixture. Unconsolidated, undrained triaxial tests were carried out in order to study the mechanical properties of the polypropylene fiber-reinforced FAS mixture and evaluate the impact of the fiber on the shear strength of the FAS mixture. It was found that the fiber length of 12 mm could significantly improve the shear strength of the polypropylene fiber-reinforced FAS mixture, and little effect is shown on the shear strength while using a fiber length of 24 mm. Additional fibers enhance the energy absorption capacity of the FAS specimens, and therefore the highest energy absorption capacity occurs when the fiber content is 1% and the fiber length is 12 mm. The peak deviator stress is enhanced impressively with the addition of polypropylene fiber. The impact of fiber on the peak deviator stress is the largest when the fiber content is within 1.0%. The fiber length has little effect on the peak deviator stress when it exceeds 12 mm.

Du et al. [10] contributed an article entitled “Feasibility Study of Grinding Circulating Fluidized Bed Ash as Cement Admixture”. With the widespread application of circulating fluidized bed (CFB) combustion technology, the popularity of CFB ash (CFBA) has increased dramatically, and its production and large-scale utilization have become increasingly important. In the context of carbon neutrality peaking, using CFBA as a cement admixture as an effective method of resource utilization not only reduces the pressures caused by carbon emissions in the cement industry but also solves the environmental problems caused by CFBA depositing. However, the formation conditions of CFBA are worse than those of traditional pulverized coal boilers. CFB ash is the combustion product of coal at 850 °C–950 °C, and the characteristics of CFBA usually include a loose and porous structure with many amorphous substances. Furthermore, it has the disadvantages of large particle size, high water-demand ratio, and low activity index when it is directly used as a cement admixture. In this study, CFBA (including fly ash (CFBFA) and bottom ash (CFBBA)) produced by a CFB boiler without furnace desulfurization with limestone was used as a cement admixture material, and the effect of grinding on the fineness, water-demand ratio, and activity index of CFBA were studied. The experimental results showed that the grinding effect could significantly reduce the fineness and water-demand ratio of CFBA as a cement mixture and improve the activity index. With the increase in the grinding time, the water-demand ratio of CFBA first decreased and then increased. CFBBA ground for 10 min and CFBFA ground for 4 min can reduce the water-demand ratio of CFBA by up to 105% and increase the compressive strength of 28-day-old CFBA cement by 7.05%. The grinding process can ensure that CFBA meets the Chinese standards for a cement admixture and realize the resource utilization of CFBA.

Pan et al. [11] provided an article entitled “The Discrepancy between Coal Ash from Muffle, Circulating Fluidized Bed (CFB), and Pulverized Coal (PC) Furnaces, with a Focus on the Recovery of Iron and Rare Earth Elements”. Coal ash (CA) is not only one of the most solid wastes from combustion, easily resulting in a series of concerns, but it is also an artificial deposit with considerable metals, such as iron and rare earth. The variation in the coal ash characteristics due to the origins, combustion process, and even storage environment has been hindering the metal utilization from coal ash. In this study, three ash samples from a lab muffle, a circulating fluidized bed (CFB), and a pulverized coal (PC) furnace were derived for the discrepancy study from the combustion furnace, including properties, iron, and rare earth recovery. The origins of the coal feed samples had more of an effect on their properties than combustion furnaces. Magnetic separation was suitable for coal ash from PC because of the magnetite product, and the iron content was 58% in the Mag-1 fraction, with a yield of 3%. The particles in CA from CFB appeared irregular and fragmental, while those from PC appeared spherical with a smooth surface. The results of sequential chemical extraction and observation both indicated that the aluminosilicate phase plays an essential role in rare earth occurrence. Rare earth in CA from muffling and CFB is facilely leached, with a recovery of approximately 50%, which is higher than that from PC ash. This paper aims to offer a reference to easily understand the difference in metal recovery from coal ash.

Volkov et al. [12] submitted an article entitled “Study of Forms of Compounds of Vanadium and Other Elements in Samples of Pyrometallurgical Enrichment of Ash from Burning Oil Combustion at Thermal Power Plants”. The results of the processing of ash from the combustion of fuel oil after roasting with the addition of Na_2_CO_3_ followed by aluminothermic melting are presented. As a result, metallic nickel and vanadium slag were obtained. Studies of slag, metal, and deposits on the electrode were carried out. The resulting metal contains about 90 wt% Ni. The main phases of scurf on the electrode are a solid solution based on periclase (Mg_1–x–y–z_Ni_x_Fe_y_V_z_O), sodium-magnesium vanadate (NaMg_4_(VO_4_)_3_), and substituted forsterite (Mg_2–x–y_Fe_x_Ni_y_SiO_4_). The processing of ash made it possible to significantly increase the concentration of vanadium and convert it into more soluble compounds. The amount of vanadium increased from 16.2 in ash to 41.4–48.1 V_2_O_5_ wt% in slag. The solubility of vanadium was studied during aqueous leaching and in solutions of H_2_SO_4_ and Na_2_CO_3_. The highest solubility of vanadium was seen in H_2_SO_4_ solutions. The degree of extraction of vanadium into the solution during sulfuric acid leaching of ash was 18.9%. In slag, this figure increased to 72.3–96.2%. In the ash sample, vanadium was found in the form of V^5+^, V^4+^ compounds, vanadium oxides VO_2_ (V^4+^), V_2_O_5_ (V^5+^), and V_6_O_13_, and nickel orthovanadate Ni_3_(VO_4_)_2_ (V^5+^) was found in it. In the slag sample, vanadium was in the form of compounds V^5+^, V^4+^, V^3+^, and V^(0÷3)+^; V^5+^ was presented in the form of compounds vanadate NaMg_4_(VO_4_)_3_, NaVO_3_, and CaxMg_y_Na_z_(VO_4_)_6_; V^3+^ was present in spinel (FeV_2_O_4_) and substituted karelianite (V_2–x–y–z_Fe_x_Al_y_Cr_z_O_3_). In the obtained slag samples, soluble forms of vanadium were found due to the presence of sodium metavanadate (NaVO_3_), a phase with the structure of granate Ca_x_Mg_y_Na_z_(VO_4_)_6_ and (possibly) substituted karelianite (V_2–x–y–z_Fe_x_Al_y_Cr_z_O_3_). In addition, spinel phases of the MgAl_2_O_4_ type beta-alumina (NaAl_11_O_17_), nepheline (Na_4–x_K_x_Al_4_Si_4_O_16_), and lepidocrocite (FeOOH) were found in the slag samples.

## 3. Summary and Outlook

This Special Issue of *Materials* was well-supported by numerous submissions and the final publication consists of twelve high-quality peer-reviewed articles. It is anticipated that due to this success, a new Special Issue (“Advances in Mineral Processing, Waste Recycling and Extractive Metallurgy”, website: https://www.mdpi.com/journal/materials/special_issues/DLW8U77F3I accessed on 20 December 2022) will be commissioned as a follow-up to accept global contributions from the hydrometallurgy and mineral processing community.
